# Supplementary material for: Sex Differences in the Association between Risk of Anterior Cruciate Ligament Rupture and COL5A1 Polymorphisms in Elite Footballers
Source: Genes (Basel). 2022 Dec 22;14(1):33. doi: 10.3390/genes14010033 (PMC9858943; doi:10.3390/genes14010033)
Supplement: Supplementary file 1 [file genes-14-00033-s001.zip › TableS1.pdf]

Table S1: The list of the SNPs, and their genomic position and location within each candidate gene					
SNP ID	CHROMOSOME	POSITION	EFFECT	LOCATION	GENES
rs1126499	X	153506051	Missense / Synonymous	Coding / Promoter	BGN
rs1052576	1	15506048	Missense	Coding / 3' UTR	CASP9
rs2076696	1	40306301		Promoter	COL9A2
rs7533552 (rs2228564)	1	40307477	Missense	Coding Region	COL9A2
rs12077871	1	40307478	Stop gained / Missense	Coding region	COL9A2
rs1676486	1	102888582	Missense	Coding region / 3' UTR	COL11A1
rs3753841	1	102914362	Missense	Coding region / 3' UTR	COL11A1
rs1049434	1	112913924	Synonymous / Missense	Coding region	SLC16A1
rs17602729	1	114693436	Stop gained / Splicing region / Missense	Coding region	AMPD1
rs2228145	1	154454494	Missense	Coding region	IL6R
rs142349285	1	161983875	Missense	Coding region	OLFML2B
rs763110	1	172658358		Promoter<br>Intergenic	FASLG
rs1800872	1	206773062		Promoter / 5' UTR<br>5' UTR	IL10<br>IL19
rs1800896	1	206773552		Promoter / 5' UTR<br>5' UTR	IL10<br>IL19
rs28932472	2	25161179	Missense	Coding region	POMC
rs2289360	2	27079297		Intron	EMILIN1
rs2287037	2	102362568		Intron	IL18R1
rs1420106	2	102418584		Promoter<br>Promoter<br>Intergenic	IL18RAP<br>IL18R1
rs1420100	2	102420542		Intron	RASGRP3
rs917997	2	102454108		Promoter<br>Promoter<br>Intergenic	IL18R1<br>IL18RAP
rs1800587	2	112785383		Promoter<br>Intergenic	IL1A
rs1143627	2	112836810		Promoter / 5' UTR	IL1B
rs16944	2	112837290		Promoter<br>Intergenic	IL1B
rs2742327	2	178722960		Intron	TTN
rs1800255	2	188999354	Missense	Coding region	COL3A1
rs3834129	2	201232809		Promoter<br>Intergenic	CASP8
rs1045485	2	201284866	Missense	Coding region	CASP8
rs9290271 (rs2595908)	3	100997976			
rs1870377	4	55106807	Missense	Coding region	KDR
rs2071559	4	55126199		Promoter<br>Intergenic	KDR
rs1011814	5	44335718		Intron	LOC339975
rs900379	5	44369554		Intron<br>Intron	FGF10<br>LOC339975
rs331079	5	128435112		Intron	FBN2
rs1799907	6	33185058	Splicing region / Splicing polypyrimidine tract		COL11A2
rs699947	6	43768652		Promoter<br>Intergenic	VEGFA

rs1570360	6	43770093		Promoter<br>Intergenic	VEGFA
rs2010963	6	43770613		5’ UTR / Promoter<br>Promoter	VEGFA<br>POLR1C
rs970547	6	75087586	Missense	Coding region	COL12A1
rs240736	6	75138465	Missense	Coding region	COL12A1
rs11154027	6	121460244		Intergenic	
rs2234693	6	151842200		Intron	ESR1
rs9340799	6	151842246		Intron	ESR1
rs9406328	6	169234915	Splicing region / Splicing polypyrimidine tract	Intron<br>Intron	THBS2<br>THBS2-AS1
rs1800795	7	22727026		Intron<br>Promoter / Intron	IL1-AS1<br>IL6
rs1011694	7	81703677			
rs5745697	7	81728033			
rs5745678	7	81742731		3’ UTR	HGF
rs10263021	7	148228400		Intron	CLIC1
rs1800972	8	6877901		5’ UTR / Promoter	DEFB1
rs13317	8	38411996		3’ UTR	FGFR1
rs4143245	9	114270742	Synonymous	Coding region / 3’ UTR	COL27A1
rs1249744	9	114281072			
rs946053	9	114287611		Intron	COL27A1
rs13321	9	115030304	Missense	Coding region<br>Intron	TNC<br>DELEC1
rs2104772	9	115046506	Missense	Coding region<br>Intron	TNC<br>DELEC1
rs13946	9	134842386		3’ UTR	COL5A1<br>LOC101448202
rs146776422	9	134842492		3’ UTR	COL5A1<br>LOC101448202
rs55748801	9	134842493		3’ UTR	COL5A1<br>LOC101448202
rs12722	9	134842570		3’ UTR	COL5A1<br>LOC101448202
rs3196378	9	134843036		3’ UTR	COL5A1<br>LOC101448202
rs71746744<br>(rs10628678)	9	134843172		3’ UTR	COL5A1<br>LOC101448202
rs16399	9	134843387		3’ UTR	COL5A1<br>LOC101448202
rs1134170	9	134843428		3’ UTR	COL5A1<br>LOC101448202
rs4919510	10	100975021		Intron<br>Promoter	SEMA4G<br>MIR608
rs680	11	2132404		3’ UTR	IGF2
rs3213221	11	2135814		Promoter<br>Intron	MIR483<br>IGF2
rs1815739	11	66560624	Synonymous / Stop gained	Coding region<br>Promoter	ACTN3<br>CTSF
rs12574452	11	69816963		Intron	HNRNPF
rs11225395	11	102725749		Promoter	MMP8
rs486055	11	102779693	Missense	Coding region	MMP10
rs1144393	11	102798678		Promoter	MMP1
rs1799750	11	102799765		Promoter<br>Intron	MMP1<br>WTAPP1

rs650108	11	102838056		Intron	MMP3
rs679620	11	102842889	Stop gained / Missense	Coding region	MMP3
rs2276109	11	102875061		Promoter<br>Intergenic	MMP12
rs731236	12	47844974	Synonymous	Coding region / 3' UTR	VDR
rs2228570	12	47879112	Start lost	Coding region /<br>Promoter	VDR
rs11613457	12	57618450		Promoter	SLC26A10
rs2268578	12	91107421			
rs516115	12	91163515		Intron	DCN
rs13312816	12	91181430		Promoter / Intron	DCN
rs2761884	14	53954334		Intron	BMP4
rs11549467	14	61740857	Missense	Coding region	HIF1A
rs4903399	14	76308859		Promoter	ESRRB
rs7157192	14	76400617		Intron	ESRRB
rs17583842	14	76514762			
rs367819510	14	94442773	Missense	Coding region	SERPINA11
rs2073711	15	65201874	Missense / Splicing region	Coding region	CILP
rs199820704	15	80175019	Missense	Coding region /<br>Promoter	FAH
rs2351491	15	88854874	Synonymous	Coding region	ACAN
rs1042631	15	88859008	Synonymous	Coding region	ACAN
rs1516797	15	88867083		Intron	ACAN
rs243865	16	55477894		Promoter	MMP2
rs2285053	16	55478465		Promoter	MMP2
rs4362400	16	77939459			
rs4227	17	7587859		3' UTR	MPDU1
rs2857656	17	34254988		Promoter<br>Intergenic	CCL2
rs1800012	17	50200388		Promoter	COL1A1
rs1107946	17	50203629		Promoter<br>Promoter	LINC01969<br>COL1A1
rs820218	17	75691415		Intron	SAP30BP
rs2279115	18	63319604		Promoter / 5' UTR<br>Promoter	BCL2<br>KDSR
rs1559186	19	9996260	Synonymous / Splicing region	Coding region	COL5A3
rs143383	20	35438203		5' UTR / Promoter	GDF5<br>LOC109461476
rs3918242	20	46007337		Promoter<br>Intergenic	MMP9<br>NA
rs17576	20	46011586	Missense	Coding region	MMP9
rs61734651	20	62819980	Missense / Splicing region	Coding region	COL9A3
rs151058	21	26939253		Intron	ADAMTS5
rs162502	21	26960687		Intron	ADAMTS5
